# A Novel Aspect of Tumorigenesis—BMI1 Functions in Regulating DNA Damage Response

**DOI:** 10.3390/biom5043396

**Published:** 2015-12-01

**Authors:** Xiaozeng Lin, Diane Ojo, Fengxiang Wei, Nicholas Wong, Yan Gu, Damu Tang

**Affiliations:** 1 Department of Medicine, Division of Nephrology, McMaster University, Hamilton, ON L8S 4L8, Canada; E-Mails: linx36@mcmaster.ca (X.L.); ojod@mcmaster.ca (D.O.); wongnyh@gmail.com (N.W.); yangu@hotmail.ca (Y.G.); 2 Father Sean O’Sullivan Research Institute, Hamilton, ON L8N 4A6, Canada; 3 The Hamilton Center for Kidney Research, St. Joseph’s Hospital, Hamilton, ON L8N 4A6, Canada; 4 The Genetics Laboratory, Longgang District Maternity and Child Healthcare Hospital, Longgang District, Shenzhen 518000, China; E-Mail: haowei727499@163.com

**Keywords:** BMI1, histone ubiquitination, DNA damage response (DDR), ATM, γH2AX, H2A, repair of double-stranded DNA breaks, checkpoint activation

## Abstract

BMI1 plays critical roles in maintaining the self-renewal of hematopoietic, neural, intestinal stem cells, and cancer stem cells (CSCs) for a variety of cancer types. BMI1 promotes cell proliferative life span and epithelial to mesenchymal transition (EMT). Upregulation of BMI1 occurs in multiple cancer types and is associated with poor prognosis. Mechanistically, BMI1 is a subunit of the Polycomb repressive complex 1 (PRC1), and binds the catalytic RING2/RING1b subunit to form a functional E3 ubiquitin ligase. Through mono-ubiquitination of histone H2A at lysine 119 (H2A-K119Ub), BMI1 represses multiple gene loci; among these, the *INK4A/ARF* locus has been most thoroughly investigated. The locus encodes the p16INK4A and p14/p19ARF tumor suppressors that function in the pRb and p53 pathways, respectively. Its repression contributes to BMI1-derived tumorigenesis. BMI1 also possesses other oncogenic functions, specifically its regulative role in DNA damage response (DDR). In this process, BMI1 ubiquitinates histone H2A and γH2AX, thereby facilitating the repair of double-stranded DNA breaks (DSBs) through stimulating homologous recombination and non-homologous end joining. Additionally, BMI1 compromises DSB-induced checkpoint activation independent of its-associated E3 ubiquitin ligase activity. We review the emerging role of BMI1 in DDR regulation and discuss its impact on BMI1-derived tumorigenesis.

## 1. Introduction

The *BMI1* (B lymphoma Mo-MLV insertion region 1) gene was identified as a collaborating oncogene with *Myc* in the tumorigenesis of B cell lymphoma in 1991 [1,2]. This initial research set the tone for subsequent investigations in the last two decades that collectively demonstrated BMI1’s multiple roles in promoting oncogenesis. The Polycomb group protein BMI1 is a component of the Polycomb repressive complex 1 (PRC1). BMI1 stimulates the E3 ubiquitin ligase activity of PRC1 via binding and stabilizing the catalytic subunit RING2/RING1b [3]. It also plays a major role in PRC1-catalyzed mono-ubiquitination of histone H2A at lysine (K) 119 (H2A-K119Ub) [3,4,5,6]. This chromatin modification is well established for silencing gene expression [7,8]. BMI1-associated E3 ubiquitin ligase activity represses multiple gene loci; among those, the *INK4A/ARF* locus is important to oncogenesis [9,10]. The locus encodes two critical tumor suppressors (p16INK4A and human p14ARF/mouse p19ARF) that activate the pRb and p53 pathways, respectively [11,12]. BMI1 is upregulated in a variety of cancer types, including lymphomas [13,14,15], prostate cancer [16], non-small cell lung cancer (NSCLC) [17], colon cancer [18], breast cancer [19], and nasopharyngeal carcinoma [20]. This upregulation occurs concurrently with a downregulation of INK4A and ARF in prostate cancer, NSCLC, and colon cancers [16,17,18], supporting the notion that repression of the *INK4A/ARF* locus contributes to BMI1-stimulated tumorigenesis.

The concept is also supported by pre-clinical investigations. Transgenic expression of BMI1 specific in the lymphoid compartment was sufficient to induce lymphomagenesis in 14% of mice expressing high levels of the transgene and significantly enhanced lymphomagenesis in combination with Eμ-myc [21]. The *N*-terminal ring-finger (RF) domain of BMI1 is required for this lymphomagenesis [22]; RF mediates BMI1 association with the catalytic subunit RING2/RING1b of PRC1 and thus is essential for the E3 ubiquitin ligase activity [3,4,5,6], indicating the involvement of the ligase activity-mediated suppression of the *INK4A/ARF* locus in BMI1-contributed lymphomagenesis. Indeed, BMI1 deficiency leads to elevated expression of *INK4A/ARF* in the lymphoid organs, which induced apoptosis likely through p53 stabilization; importantly, deletion of the *INK4A/ARF* locus rescued the apoptosis [10]. BMI1 also plays an essential role in the tumorigenesis of neuroblastoma in a process that is associated with a robust decrease in p16INK4A [23]. In a mouse model for intestinal adenocarcinoma, BMI1^−/−^ mice were protected from developing intestinal adenocarcinoma though ARF-dependent p53 activation [24]. Additionally, BMI1 is required for the self-renewal of adult hematopoietic and neural stem cells in part by suppressing the *INK4A/ARF* locus [25,26,27,28,29,30], and for the maintenance of a subpopulation of intestinal stem cells (ISC) [31,32]. The physiological functions of BMI1 in maintaining the self-renewal of stem cells likely contribute to its role in sustaining the self-renewal of cancer stem cells (CSCs) for lymphoma, neuroblastoma, and intestinal cancers [33]. Likewise, BMI1 plays a critical role in the self-renewal of prostate and lung stem cells [34,35], and thus makes an essential contribution to their cancer stem cells [33].

It is apparent that BMI1 also uses other pathways. For instance, BMI1 plays a critical role in the self-renewal of NSC during development through inhibition of p21^CIP1^ [36]; BMI1 regulates the self-renewal of ISCs through suppression of the *INK4A/ARF* locus and at the same time, both the Notch and Wnt pathways regulate BMI1 expression in this process [37]. BMI1 stimulates glioma through an INK4A/ARF-independent pathway [38] and the same situation also applies to BMI1/Ras-elicited hepatic carcinogenesis [39]. Upregulation of cyclin E contributes to BMI1-promoted neuroblastoma progression [40]. At the molecular level, BMI1 inhibits PTEN function and collaborates with TWIST to induce epithelial-mesenchymal transition (EMT) and metastasis [41,42]. BMI1 has also been reported to enhance telomerase activity in mammary epithelial cells and prostate cancer cells [43,44].

While BMI1 suppresses *PTEN* gene expression in nasopharyngeal epithelial cells [41], PTEN is able to inhibit BMI1 function via a physical association [44]. Intriguingly, this association does not require PTEN’s PIP3 [phosphatidylinositol (3,4,5)-trisphosphate] phosphatase activity and occurs inside the nucleus [44]. Nuclear PTEN plays an important role in facilitating DNA damage response (DDR), and thus contributes to genome stability [45,46]. These observations thus suggest a possibility that nuclear PTEN inhibits BMI1’s role in DDR regulation. Indeed, this possibility has been recently demonstrated. We will focus on the discussions of this emerging role of BMI1 in DDR regulation. For other processes contributing to BMI1-stimulated tumorigenesis, please see the elegant reviews by Siddique and Saleem and by Benetatos *et al*. [33,47].

## 2. General Aspects of DNA Damage Response

**Figure 1:**
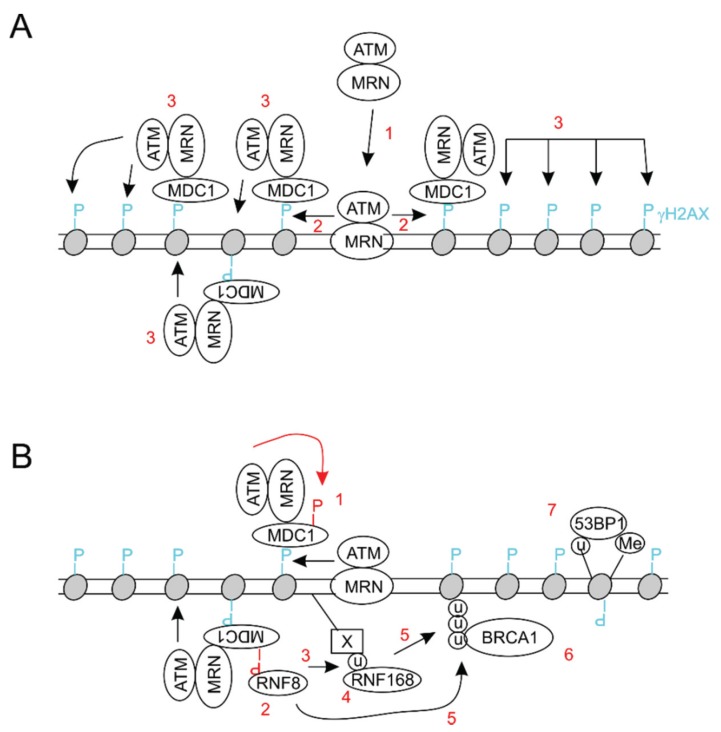
Schematic illustration of the assembly of the double-stranded DNA break (DSB) repair complex. (**A**) The MRN complex recruits ATM to DSB (1), allowing ATM to phosphorylate S139 of H2AX (γH2AX) (2); and (3): MDC1 is subsequently recruited and the CK2-phosphorylated SDT motifs at the *N*-terminus of MDC1 interact with the MRN-ATM complex, resulting in the spread of the γH2AX domain. (**B**) The association of the MRN-ATM complex with MDC1 leads to ATM-mediated phosphorylation of MDC1 (1), RNF8 recruitment (2), RNF8-derived ubiquitination of unknown substrate (X) (3), and the binding of RNF168 (4); and (5): RNF8 and RNF168 coordinately ubiquitinate histone H2A/H2AX, paving the way for BRCA1 recruitment (6). The ubiquitination of H2A K15 together with H4 K20 methylation contribute to 53BP1 recruitment.

DDR is the central mechanism to maintain genome integrity and to faithfully transmit genetic codes to the next cell generation. In the presence of a variety of DNA lesions, a complex network of DDR is initiated by three apical PI3 kinase related kinases (PIKKs), ATM (ataxia-telangiectasia mutated), ATR (ATM- and Rad3-related), and DNA-PK. Their actions coordinate two major DDR processes: checkpoint activation to stop cell proliferation and repair mechanism preparation to restore DNA integrity [48]. While ATM and DNA-PKcs (CS: catalytic subunit) are activated by double-stranded DNA breaks (DSBs), ATR is primarily activated by single-stranded DNA breaks [48,49]. DSBs are the most toxic form of DNA lesions; as a result, the cell has developed a sophisticated response system to control DSB-derived toxicity. At the center of these systems lies ATM. Activation of ATM induces checkpoint activation and initiates DSB repair through homologous recombination (HR) [48,50]. ATM achieves these tasks by phosphorylation of a set of downstream substrates, including CHK2 and histone H2AX. Phosphorylation of CHK2 at threonine (T) 68 activates the kinase, which contributes to checkpoint activation; phosphorylation of H2AX at serine 139 (γH2AX) provides a docking site to assemble the repair complex in the DSB surrounding regions for DSB repair [48,51,52]. This process is initiated when the MRN (Mre11-Rad50-NBS1) complex directly binds DSBs through the MR components [53], leading to ATM recruitment to DSBs through its direct interaction with NBS1 [53] (Figure 1A1). ATM then produces γH2AX (Figure 1A2) to which the MDC1 (mediator of DNA damage checkpoint protein 1) protein is recruited [54,55]. The *N*-terminal MDC1 has several SDT (serine-aspartic acid-threonine) repeats which are constitutively phosphorylated by casein kinase 2 [56,57,58]; their phosphorylation enables the association of the ATM/MRN complex through the phosphoprotein binding domain of NBS1 [59,60]. This leads to further production of γH2AX (Figure 1A3) and these repetitive events result in the spread of γH2AX along a long stretch of DNA region surrounding DSB (Figure 1A3). ATM phosphorylates MDC1, which creates a binding site for the RNF8 E3 ubiquitin ligase [61,62,63]. The ligase activity of RNF8 is required for the formation of ubiquitin chains, which recruits the RNF168 E3 ubiquitin ligase [64,65,66]. Although it was suggested that mono-ubiquitination of histone H2A by RNF8 creates the binding site for RNF168 recruitment [67], new evidence indicates that RNF8 induces the ubiquitin conjugation on as yet unidentified substrates, leading to RNF168 recruitment which then initiates the mono-ubiquitination of H2A/H2AX at K13 and K15 [68]. It is likely that RNF8 subsequently extends the H2A/H2AX ubiquitination produced by RNF168 [68]. Collectively, the concerted enzymatic activities of RNF8 and RNF168 conjugate ubiquitin to histone H2A and γH2AX, which provide the binding sites for BRCA1 [64] (Figure 1B). BRCA1 plays a critical role in the commitment of the cell to homologous recombination (HR)-mediated DSB repair [69].

H2A ubiquitination also sets a stage to repair DSBs through non-homologous end joining (NHEJ) (Figure 1B7). The recruitment of 53BP1 into DSBs is a critical step to commit cells to repair DSBs using NHEJ but not HR [69,70,71]. The recruitment is mediated by the association of 53BP1’s tandem tudor domains with H4K20me2 (histone H4 lysine 20 dimethylation) [72], and is also regulated by RNF8/RNF168 [63,64]. For example, knockdown of RNF8 inhibited the retention of 53BP1 at DSB [61,62,63]. In support of these observations, a *C*-terminal ubiquitylation-dependent recruitment (UDR) domain was recently identified in 53BP1 and UDR contributes to the recruitment of 53BP1 to DSBs through specific binding to H2A K15 ubiquitination [73].

## 3. BMI1 Enhances DSB Repair by Promoting Histone H2A and γH2AX Ubiquitination

Accumulative evidence clearly demonstrates the critical functions of histone ubiquitination in DSB repair through HR and NHEJ. In agreement with this concept, BMI1 has been recently reported to play a role in histone H2A and H2AX ubiquitination through the BMI1/RIN1b E3 ubiquitin ligase, and thus contributes to DSB repair.

PRC1 affects chromatin structure through ubiquitination of histone H2A at K119 using the BMI1/RING1b E3 ubiquitin ligase [3,4,5,6]. This is a well demonstrated mechanism in suppressing gene transcription; additionally, recent developments add a new role for this modification in facilitating DSB repair. BMI1 was detected being rapidly recruited to DNA lesions caused by local micro-irradiation using UV laser beam, ionizing irradiation (IR), and hydroxyurea (HU) in a set of cell types, including U2OS, mouse embryonic fibroblasts (MEFs), HeLa, and CD133+ glioblastoma multiforme (GBM) cells [74,75,76,77,78]. The recruitment of the BMI1/RING1b E3 ubiquitin ligase is required for the mono-ubiquitination of γH2AX and H2A likely at K119 in the DSB regions in U2OS cells and MEFs, as downregulation of BMI1 abolished the modification [74,76]. In agreement with histone ubiquitination being critical in DSB repair, the presence of BMI1 in IRIF (ionizing radiation-induced foci) contributes to DSB repair. For incidence, BMI1 deficient MEFs display a two-fold reduction in repair of DSBs induced by calicheamicin (CLM) at 5 h post treatment and the defects are rescued upon re-expression of BMI1 [74]. Furthermore, BMI1 downregulation compromises the survival of U2OS, HeLa, and GBM cells with respect to IR treatment [74,75,76,77], likely results from a reduction in DSB repair due to BMI1 downregulation, thereby indirectly supporting the notion that BMI1 facilitates DSB repair.

BMI1 enhances DSB repair at least in part through HR. Using an I-Scel-based *in vivo* HR assay in 293T cells, knockdown of BMI1 reduced HR-mediated DSB repair [78], an observation that is in accordance with BMI1-facilitated BRCA1 recruitment [74]. BRCA1 is essential for the commitment of cells to repair DSB using HR [69,79]. Nonetheless, evidence also supports a role of BMI1 in promoting non-homologous end joint (NHEJ)-mediated DSB repair. For example, NHEJ requires the recruitment of 53BP1 to DSBs [69,79]. BMI1 enhances this recruitment and may physically interact with 53BP1 [74,75]. Direct evidence supporting a major role of BMI1/RING1b in promoting NHEJ was obtained under the situation of dysfunctional telomere-initiated NHEJ. Knockdown of either RING1b or BMI1 significantly reduced NHEJ-mediated telomere fusion [80]. However, RING1b deficiency in MEFs did not affect the repair of DSBs caused by gamma irradiation and only transiently decreased NHEJ-derived DSB repair at heterochromatin loci [80]. In aggregate, evidence supports the contributions of BMI1-assocaited E3 ubiquitin ligase to DSB repair [81].

Additionally, the involvement of the ligase activity in DSB repair is supported by the requirement of RF domain for BMI1 recruitment to DSBs [74,76]. RF mediates BMI1 association with the catalytic subunit RING2/RING1b, and is thus essential for BMI1-associated E3 ubiquitin ligase activity [6,82]. With this knowledge in mind, it might be worth determining whether re-expression of the RF-deleted BMI1 mutant is able to rescue the defects in repairing CLM-caused DSB in BMI1^−/−^ MEFs; this will provide an additional support that the observed rescue using wild type BMI1 is attributable to its-associated E3 ubiquitin ligase activity [74].

Structural analysis also supports the concept that BMI1-associated ligase activity is important to its role in facilitating DSB repair [68]. The RNF168 and BMI1/RING1b, but not RNF8, E3 ubiquitin ligases are able to conjugate ubiquitin to the nucleosomal contents of H2A/H2AX, an activity that is attributable to the positively charged residues R57 in RNF168 and K93 in RING1b; whereas the conservative residue for RNF8 is a negatively charged residue D443 [68]. Substitution of either R57 of RNF168 or K93 of RING1b to a negatively charged D residue abolishes their ability to ubiquitinate nucleosomal H2A/H2AX at K13/15 for RNF168 and K118/K119 for BMI1/RING1b, respectively [68]. On the substrate side, H2A/H2AX contains a nucleosome acidic patch (E61, D90, and E92) that is required for both E3 ubiquitin ligases to ubiquitinate the respective lysine residues [83,84]. In support of these observations, a recent crystallized structure of BMI1/RING1b revealed that the structural interface of RING1b for the H2A nucleosome acidic patch included R98, K93, and K97 with R98 being most critical [85]. R98 inserted into the acidic pocket formed by E61, D90, and D92 of H2A, and made contacts to each of the side chains [85]. Consistent with these structural roles, substitution of R98 with alanine led to a 50-fold decrease in nucleosomal ubiquitination and affinity to bind nucleosome [85]. In comparison to the dominant role of RING1b in PRC1’s association with nucleosome, BMI1 does not make a significant contribution to the nucleosome binding of PRC1 [85]. While it is likely that these structural details are involved in BMI1/RING1b-derived ubiquitination of H2A/H2AX at K118/K119 under DDR, this has yet to be demonstrated.

It also remains unclear what proportion of DSB repair is contributed by BMI1-associated E3 ubiquitin ligase activity. Nonetheless, it seems that a significant proportion occurs without a major contribution from BMI1. Although BMI1^−/−^ MEFs contain 2-fold more DSBs during a course of 5-h repair of CLM-induced DSBs in comparison to control cells, approximately 60% of DSBs in BMI1^−/−^ MEFs are repaired [74]. Will more DSBs be repaired in BMI1^−/−^ MEFs if sufficient time is given? This seems likely, as knockdown of BMI1 in U2OS and HeLa cells only modestly reduced cell survival in response to IR exposure [74,76]. In support of this possibility, DSB repair in BMI1^−/−^ MEFs was only delayed compared to wild type MEFs [80] and the same was also reported in U2OS cells [86]. Alternatively, there might be factors waiting to be discovered in regulating BMI1-facilitated DSB repair. AKT has been shown to phosphorylate BMI1, which contributes to BMI1’s ability to cause the accumulation of mono-ubiquitinated H2A in IRIF in MEFs treated with a UV laser scissors [87]. Inhibition of AKT activation was without effects on either BMI1 recruitment to DSBs or the recruitment of 53BP1, indicating that NHEJ-mediated DSB repair may not be dramatically affected [87].

## 4. The BMI1/RING1b E3 Ubiquitin Ligase Contributes to DDR-Induced Transcription Repression

Transcription poses a threat to genome integrity [88] and the threat is controlled by the DDR machinery [89]. Of note, DSBs are able to repress transcription occurring in the flanking regions, a process that depends on ATM and H2A K119 monoubiquitination [90]. This post-translational modification is well recognized for repression of promoter activity, and is mediated by the BMI1/RING1b E3 ubiquitin ligase [3], suggesting that BMI1/RING1b plays a role in producing H2A K119 monoubiquitination in the active transcription regions near DSB. Indeed, knockdown of BMI1 significantly decreased DSB-induced transcription repression concurrently with a significant reduction in H2A K119 monoubiquitination [86]. Mechanistically, ATM phosphorylates a transcription elongation factor ENL, causing its association with BMI1. This interaction leads to the accumulation of PRC1 at transcription elongation sites, the subsequent H2A K119 monoubiquitination, and transcription repression near DSBs [91]. It is likely that factors in addition to ENL also regulate ATM-mediated recruitment of BMI1/RING1b to the site of active transcription flanking DSBs. For example, it has been implied that BAF180 upon phosphorylation by ATM may facilitate PRC1 accumulation at the transcription sites close to DSB [86]. Collectively, evidence supports the contributions of BMI1-associated E3 ubiquitin ligase to DSB-induced transcription repression.

## 5. BMI1 Attenuates DSB-Induced Checkpoint Activation by Reducing ATM Activation

The second major aspect of DDR is checkpoint activation [48]. In response to DSBs, ATM stops cell cycle progression through phosphorylation of downstream targets, CHK2 and p53 [48]. Subsequently, CHK2 phosphorylates CDC25C at serine 216 (S216), an action that inactivates CDC25C’s ability to dephosphorylate CDC2 at tyrosine 15 (Y15) and T14. Dephosphorylation of both residues is required for CDC2 activation; CDC2 (CDK1) kinase activity is essential for cell cycle progression through the G2/M phase [92,93,94,95]. ATM phosphorylates p53 at S15, contributing to p53 stabilization; p53 in turn transactivates p21^CIP1^, resulting in cell cycle arrest [48].

The involvement of BMI1 in facilitating DSB repair thus is in accordance with its role in the regulation of checkpoint activation. Indeed, consistent with BMI1 enhancing DSB repair, BMI1 was observed to accordingly affect cell cycle progression, specifically G2/M arrest [76,78]. For example, BMI1 overexpression was observed to enhance IR-induced γH2AX in GBM cells [75].

On the other hand, ectopic expression of BMI1 can also reduce γH2AX in IR-treated normal human keratinocytes [96], while knockdown of BMI1 significantly increases γH2AX in cisplatin-treated ovarian cancer cell lines A-2780 and CP-70 [97], suggesting that BMI1 might compromise DSB-elicited checkpoint activation. In accordance with this notion, ectopic BMI1 decreased γH2AX in MCF7 breast cancer and DU145 prostate cancer cells in response to etoposide-induced DSBs, while BMI1 knockdown in both lines enhanced the γH2AX production and its nuclear foci [98]. Consistent with DSBs first recruiting the ATM/MRN complex and ATM subsequently phosphorylating H2AX at S139 (γH2AX) in the regions proximate to DSBs [52,67] (Figure 1A), overexpression and knockdown of BMI1 in MCF7 and DU145 cells respectively enhanced and reduced etoposide-induced ATM activation and the phosphorylation of CHK2 at T68, a well-known target of ATM [98]. Additionally, G2/M arrest was reduced in etoposide-treated MCF7 cells stably overexpressing BMI1 and enhanced in BMI1 knockdown MCF7 cells upon etoposide treatment [98]. These observations support the notion that BMI1 compromises etoposide-induced G2/M checkpoint activation through decreasing ATM activation at least in cancerous breast and prostate cancer cells.

Ectopic expression of BMI1 also reduced ATM activation and γH2AX production in non-cancerous MCF10A mammary epithelial cells in response to etoposide exposure [98]. Consistent with DSB-induced ATM activation, requiring its association with NBS1 [99,100,101,102], BMI1 associated with NBS1. Importantly, deletion of RF did not reduce BMI1’s affinity to bind NBS1 and did not alter BMI1’s ability to reduce ATM activation induced by etoposide, strongly indicating that BMI1 downregulates ATM activation independent of its-associated E3 ubiquitin ligase activity [98]. While it remains unclear how by interaction with NBS1, BMI1 reduces ATM activation, it can be envisaged that this interaction affects NBS1’s ability to activate ATM. Nonetheless, ectopic BMI1 confers resistance to etoposide-induced cytotoxicity in MCF7 cells [98] and this resistance does not require the presence of the RF domain (our unpublished observation). These results thus support a contribution of BMI1 to cell survival of DSBs through at least in part the attenuation of G2/M checkpoint activation.

## 6. Conclusions and Perspectives

### 6.1. Molecular Mechanisms of BMI1’s Contributions to DSB Repair

**Figure 2:**
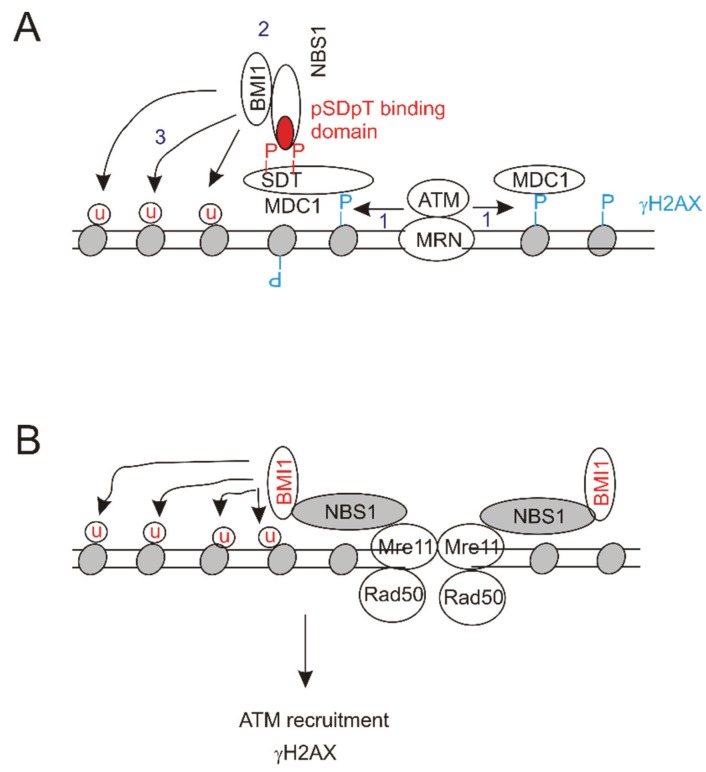
Potential pathways underlying the ubiquitination of H2A/H2AX by the BMI1-associated E3 ubiquitin ligase. (**A**) ATM (ataxia-telangiectasia mutated) is first recruited onto DSBs and then produces γH2AX (1); the phospho-SDT binding domain of NBS1 (red) associates with MDC1 along with recruiting the BM1-associated E3 ubiquitin ligase (BMI1) (2); the ligase activity then ubiquitinates histone H2A/H2AX (3); (**B**) BMI1 is recruited to DSBs via its association with NBS1 of the MRN complex, and conjugates ubiquitin to histones, which facilitates ATM activation and γH2AX production.

Histone H2A-K119Ub is a classic epigenetic mark that is associated with gene silencing [103,104]; H2A-K119Ub is also an abundant histone modification, which is produced by PRC1, and can constitute up to 10% of cellular histone H2A [3,4,6,105]. It thus remains an intriguing question whether the existing H2A-K119Ub plays a role in DSB repair, if this existing modification needs to be removed first in order to change chromatin structure to facilitate DSB repair, or other as yet identified mechanisms are present which make the existing H2A-K119Ub available for DSB repair. For the scenario of newly produced H2A-K119Ub, it may function differently from the existing one in DSB repair. This possibility is supported by experimental evidence showing that BMI1 recruitment to DSB is independent of PRC2 [74]; PRC2 action produces a docking site to recruit PRC1 to conjugate ubiquitin to H2A-K119 for gene repression [81,106]. In the situation of DSB repair, the *N*-terminal region of NBS1, containing the FHA and BRCA domains, is required for BMI1 recruitment [74]. As these domains bind the conserved phosphorylated pSer-Asp-pThr-Asp motif of MDC1 [56,107,108], this would suggest a model in which NBS1 recruits BMI1 to MDC1 (Figure 2A). In essence, this would place the BMI1 recruitment downstream of ATM action (Figure 2A), a situation that occurs under a certain setting [76].

Alternatively, BMI1 recruitment and its action may occur upstream of ATM. NBS1 almost always exists in the MRN complex and the complex directly binds DSBs via the head structure formed by Mre11 and Rad50 [53]. As NBS1 binds BMI1 [98], it is thus possible that the MRN complex recruits BMI1 directly to DSBs via NBS1 (Figure 2B). This would allow the BMI1-associated E3 ubiquitin ligase activity to conjugate ubiquitin to H2AX at K118/K119, in turn promoting ATM activation (Figure 2B). Evidence exists to support this model. Mono-ubiquitination of H2AX at K118/K119 by BMI1/RING2 enhances γH2AX following IR exposure; the γH2AX nuclear foci are significantly reduced in cells expressing the ubiquitination defective mutant H2AX (K118R/K119R) [109].

It appears that BMI1 may use different mechanisms to contribute to DSB repair (Table 1), suggesting that both models discussed above (Figure 2) can be used depending on the cellular context. The relationship between RNF8/RNF168-ubiquitinated K13/K15 and BMI1/RING1b-conjugated K118/K119 in H2A during DSB repair needs to be investigated in future research. Since the *N*-terminal K residues (K13/15) and the *C*-terminal tail K residues (K118/119) are within opposite sides of the nucleosome [68,110], both events might indeed occur independently as suggested [74].

Nonetheless, the relationship between BMI1-contributed histone ubiquitination and γH2AX and MDC1 remains unclear. The formation of the γH2AX and MDC1 complex in IRIF is well established to be an early event leading to the assembly of the DSB repair machinery [67]. It has been observed that (1) recruitment of BMI1 to DSB either depended [76] or did not depend [74] on γH2AX; (2) ATM contributed to [76] or did not play a role [75] in BMI1 recruitment to DSBs; (3) NBS1 helped to mediate [74] and was not involved [75] in the recruitment of BMI1 to DSBs; and (4) the formation of γH2AX foci was independent of [74] or was required for BMI1-mediated H2AX mono-ubiquitination [77] (Table 1). Clearly, future experiments are needed to address these issues.

**Table 1:** γH2AX nuclear foci dependent and independent BMI1 recruitment to double-stranded DNA breaks (DSBs).

Cell Type	DSB Induction	γH2AX-Mediated BMI1 Recruitment	References
MEFs ^1^	UV laser ^3^	NO	[74]
Hum fib ^2^	Ionizing radiation	NO ^4^	[75]
MEFs	UV laser	YES	[76]
HeLa	UV laser	YES ^4^	[76]
U2OS	Ionizing radiation	BMI1-mediated H2AX Ub ^5^ enhances γH2AX ^6^	[77]

^1^ mouse embryonic fibroblasts; ^2^ human fibroblasts; ^3^ UV laser scissors; ^4^ based on the impact on ATM (ataxia-telangiectasia mutated) activation; ^5^ ubiquitination; and ^6^ ATM activation and MDC1 recruitment are also enhanced.

### 6.2. Functions of BMI1 in Reducing DDR-Elicited Checkpoint Activation

Based on the contributions of BMI1 to DSB repair as discussed above, it is surprising that BMI1 reduces ATM activation in response to etoposide-induced DSBs via binding to NBS1 in a manner in which BMI1-associated E3 ubiquitin ligase activity is not required [98]. How the association results in decreased ATM activation needs to be determined in future. Will BMI1 association directly impact NBS1-derived ATM activation? NBS1 contains two functional regions that are connected by a flexible linker. The *N*-terminal phosphoprotein-binding core consists of a forkhead-associated (FHA) domain (residues 24–108), which is fused to a breast cancer *C*-terminal (BRCT) domain (residues 108–196), and a second BRCT motif (residues 221–330) [107,111,112,113]. The *C*-terminal region includes a Mre11-binding domain (residues 673–733) and an ATM-binding motif (residues 735–754) [114,115]. Mapping the BMI1-interacting regions in NBS1 will shed light on the mechanisms used by BMI1 to influence DSB-induced ATM activation.

BMI1 is well demonstrated to promote tumorigenesis. A common feature of BMI1-stimulated oncogenesis is its collaboration with multiple oncogenes. The oncogenic activities of *BMI1* were identified because of its collaboration with *c-Myc* during lymphomagenesis [1,2]. To ensure this collaboration, c-Myc directly transactivates BMI1 in leukemia, neuroblastoma, and nasopharyngeal carcinoma [116,117,118]. Additionally, BMI1 also works together with Ras [119,120], Abel [117], and hTERT [121,122,123]. However, the underlying mechanisms of these collaborations remain unspecified. Repression of the *INK4A/ARF* locus contributes to BMI1’s synergy with c-myc during tumorigenesis [10], a process in which BMI1-associated E3 ubiquitin ligase activity is required. It is also possible that BMI1 collaborates with oncogenes through inhibition of ATM; for this collaboration, the E3 ubiquitin ligase activity is not required. This property of duel oncogenic functions resembles BMI1’s involvement in DDR regulation with respect to DSB repair and checkpoint activation.

The second mechanism (ATM inhibition) responsible for BMI1’s collaboration is in good agreement with the common activation of the ATM-DDR pathway during tumorigenesis, as a tumor surveillance mechanism employed by a variety of oncogenes. This surveillance mechanism constitutes an essential anti-tumor barrier and its overcome is required for malignant transformation [124,125,126]. For example, HER2/Erb2, c-Myc, and Ras all robustly activate the ATM-mediated DDR barrier during breast cancer pathogenesis; reducing ATM’s ability to activate checkpoints allows breast cancer progression [124]. Collectively, BMI1’s ability to attenuate ATM activation independent of the E3 ubiquitin ligase activity provides a new platform by which BMI1 promotes tumorigenesis.

### 6.3. DSB Repair *vs*. Checkpoint Activation

By promoting DSB repair, BMI1 facilitates ATM activation, which will be expected to activate checkpoints, a process in which BMI1-associated E3 ubiquitin ligase activity conjugates ubiquitin to H2A/H2AX. On the other hand, BMI1 decreases ATM activation independently of the ligase activity and thus compromises checkpoint activation.

**Figure 3:**
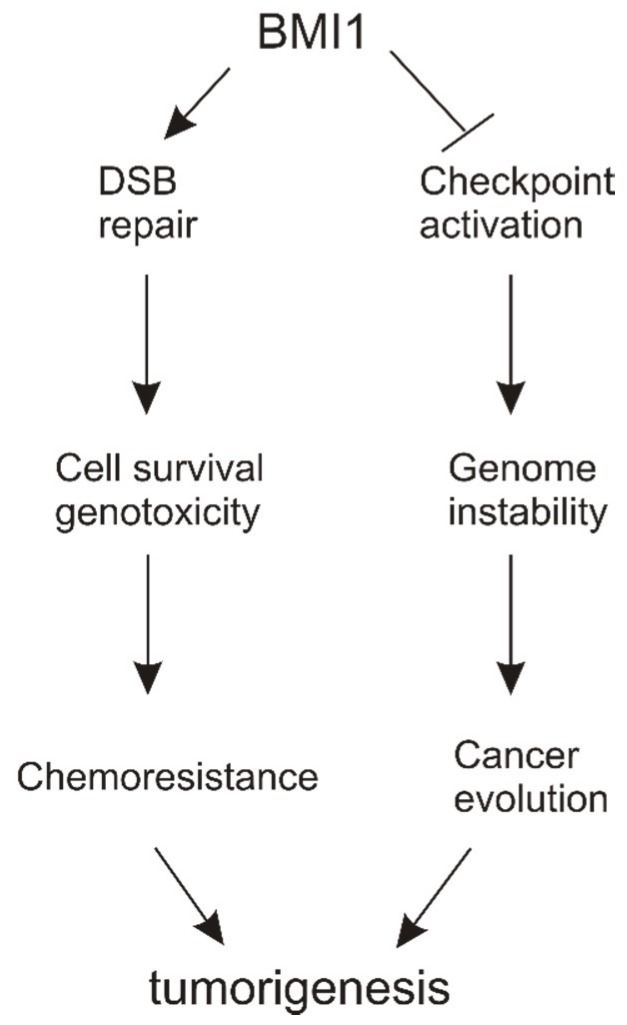
A model suggesting BMI1’s contributions to DNA damage response (DDR) regulation. BMI1 can enhance DSB repair, which plays a role in developing resistance to chemotherapy in cancer. Through reduction of checkpoint activation, BMI1 contributes to genome instability and thus cancer evolution. Both actions stimulate tumorigenesis.

While the details of how BMI1 functions in these two processes through seemingly opposite activities are still unclear, a model can be proposed (Figure 3) in which BMI1 contributes to chemoresistance in cancers by enhancing DSB repair. On the other hand, by reducing checkpoint activation, BMI1 empowers cell proliferation in the presence of DNA lesions, which contributes to genomic instability and thereby stimulating tumor progression (Figure 3).

Clearly, both models of action stimulate tumor evolution, which agrees well with BMI1’s role in tumorigenesis. However, more work is needed to provide details on the involvement of BMI1 in DDR. Nonetheless, the emerging role of BMI1 in DDR regulation is an exciting development that holds great potential for future investigations.
